# Metagenetic and Volatilomic Approaches to Elucidate the Effect of *Lactiplantibacillus plantarum* Starter Cultures on Sicilian Table Olives

**DOI:** 10.3389/fmicb.2021.771636

**Published:** 2022-02-25

**Authors:** Amanda Vaccalluzzo, Giuseppe Celano, Alessandra Pino, Francesco Maria Calabrese, Paola Foti, Cinzia Caggia, Cinzia Randazzo

**Affiliations:** ^1^Department of Agricultural, Food and Environment, University of Catania, Catania, Italy; ^2^Department of Soil, Plant and Food Science, University of Bari Aldo Moro, Bari, Italy; ^3^ProBioEtna srl, Spin-off of University of Catania, Catania, Italy

**Keywords:** 16S amplicon-based sequencing, colture-independent approach, lactobacilli, olives fermentation, VOCs

## Abstract

The present study aimed to evaluate the effect of selected *Lactiplantibacillus plantarum* strains on both microbiota composition and volatile organic compound profile of Sicilian table olives. Two mixed cultures, named O1 and O2, were set up for pilot-plan scale fermentations at 5% of NaCl. Uninoculated table olives at 5 and 8% (C5 and C8) of salt were used as control. The fermentation process was monitored until 80 days through a dual approach, which included both classical microbiological and 16S amplicon-based sequencing and volatilomics analyses. Compared with control samples (C5 and C8), experimental samples, inoculated with starter cultures (O1 and O2), exhibited a faster acidification with a more pronounced drop in pH. Metagenetics data revealed significant differences of microbiota composition among samples, highlighting the dominance of lactobacilli in both experimental samples; a high occurrence of *Enterobacter* genus only in control samples with 5% of NaCl; and the presence of *Bacteroides*, *Faecalibacterium*, *Klebsiella*, and *Raoultella* genera only in control samples with 8% of NaCl. Furthermore, microbiota composition dynamics, through the fermentation process, significantly affected the volatile organic compounds of the final products, whereas no compounds involved in off-odors metabolites were detected in all samples investigated. In conclusion, the addition of the proposed starter cultures and the use of low concentrations of sodium chloride positively affected the microbiota and volatile organic compounds, ensuring the microbiological safety and the pleasant flavors of the final product.

## Introduction

Among fermented foods, table olives are one of the most well-known and produced fermented vegetables, especially in the Mediterranean area, with a large consumption worldwide (Vaccalluzzo et al., 2020a). The high contents of vitamins, minerals, dietary fibers, short-chain fatty acids, and bioactive compounds, such as polyphenols, contribute to the high nutritional and functional value of table olives (Argyri et al., 2020). Based on data recently reported by the International Olive Council (2019), table-olives production is currently close to 2.9 million tons/season. The fermentation of table olives involves the conversion of inedible compounds into edible organic biomolecules, due to the metabolic activity of autochthonous or deliberately added starter cultures. It is already well established that in table olives a complex and variable microbial consortium, mainly composed of lactic acid bacteria (LAB) and yeasts, is present (Ashaolu and Reale, 2020; Vaccalluzzo et al., 2020a). This microbial consortium is mainly responsible for the debittering process, which occurs spontaneously in table olive fermentation, through the activity of β-glucosidase and esterase enzymes. In recent years, the approach to study the complex microbiota of table olives has been completely revolutionized, contributing to better understand its dynamism in composition and functionality. Amplicon-based metagenomics analysis targeting either 16S rRNA gene or internal transcribed spacers (ITSs) DNA region is the most widely used technique to reveal the complexity of microbial consortium of LAB and yeasts/fungal communities, respectively, in food matrices, as well as in table olives (De Filippis et al., 2017; Ferrocino and Cocolin, 2017; Vaccalluzzo et al., 2020a). Indeed, metagenetic studies allowed gaining a comprehensive view of table-olives microbiota at different taxonomic levels, revealing the presence of unexpected bacteria during fermentation. The presence of halophilic and soil-related bacteria, belonging to *Ralstonia* and *Roultella* genera, has been revealed in Nocellara Etnea and in Nyons black table olives, respectively (Cocolin et al., 2013; Penland et al., 2020). Through high-throughput bar-coded pyrosequencing analysis of ITS1-5.8 S-ITS2 region, the presence of a complex fungal consortium, including phytopathogenic, saprophytic, spoiling, and fermentative genera, never detected by using culture-dependent techniques, has been recently revealed (Vaccalluzzo et al., 2020a). Furthermore, volatilomic approaches can detect biomolecules and aromatic compounds, generated during the fermentation process. According to previous reports on fermented table olives, headspace solid-phase microextraction (HS-SPME) coupled to gas chromatography–mass spectrometry (GC–MS) is the most widely used technique to investigate the volatilome. Studies conducted by De Angelis et al. (2015) and Martorana et al. (2017) showed that the use of starter culture positively influenced the volatile organic compound (VOC) profile of fermented table olives, increasing the pleasant compounds in the final product. Cultivar and fermentation process, together with the microbial dynamics, strongly influences the profile of volatile compounds in table olives. Indeed, Nanou et al. (2021) reported a significant difference in the VOC profile between the two varieties, Halkidiki and Conservolea, fermented through the same process, and Bleve et al. (2014, 2015) highlighted differences in volatile compounds between two varieties. In addition, de Castro et al. (2019) found a correlation between metagenetics and volatilomics, identifying microbial species positively correlated with off-odors of table olives.

Up to now, few studies evaluated the possibility to set up table olives with low sea salt content and without the addition of any NaCl replacements (Randazzo et al., 2017; Pino et al., 2018a). Recently, Pino et al. (2018a,2019) demonstrated that the reduction of NaCl content to 5% did not increase the risk of microbial spoilage or the overgrowth of foodborne pathogens, allowing to obtain a safe product with appreciated sensorial traits.

The aim of the present study was to elucidate the effect of two different starter cultures containing *Lactiplantibacillus plantarum* strains on the microbiota composition and organic compound profile of Sicilian table olives, processed at 5 and 8% of salt content. The study was conducted using a dual approach that includes both conventional and amplicon-based metagenetics analyses and volatilomics analyses.

## Materials and Methods

### Olive-Processing Method

In the present study, olives of Nocellara Etnea cultivar, kindly provided by local companies, situated in Adrano and Paternò, Catania, Sicily, were processed at industrial scale, following the Sicilian style method, without the addition of sodium hydroxide solution. After harvesting (September–October, 2019), drupes were pretreated according to Pino et al. (2018a) and directly placed into brines, containing 5 and 8% of sea salt.

### Inoculum of Selected *Lactiplantibacillus plantarum* Strains

**FIGURE 1:**
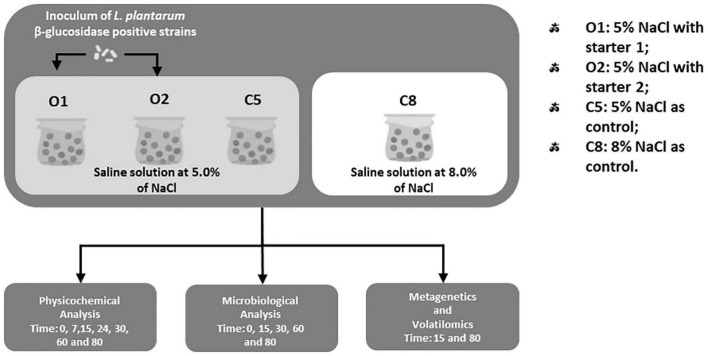
Study design of experimental table olives of Nocellara Etnea cv.

The *L. plantarum* C11C8, F1.16, and F3.5 strains, previously isolated from brine samples of natural Sicilian table olives at 5% of NaCl (Pino et al., 2019) and characterized for the ability to grow and to degrade the oleuropein under stress conditions (Vaccalluzzo et al., 2020b), were used as starter cultures. In detail, the two starter cultures were set up as follows: starter 1: with *L. plantarum* F1.16 and F3.5 strains and starter 2: with *L. plantarum* C11C8, F1.16, and F3.5 strains. The starter cultures were inoculated to a final cell density of 7 log CFU/mL, directly after brining. As displayed in Figure 1, four different fermentations were carried out: O1: 5% of NaCl, with the addition of starter 1; O2: 5% of NaCl, with the addition of starter 2; and C5 (5% of NaCl) and C8 (8% of NaCl) without the addition of any starters, both used as control. All fermentations were carried out at room temperature (18 ± 2°C) and monitored for 80 days. In addition, salt was periodically added into the samples, in order to maintain the initial sodium chloride concentration. Each fermentation was carried out in triplicate.

### Chemical and Microbiological Analyses

The pH of brine was measured, during the fermentation process at times 0,7,15, 24, 30, 60, and 80 days, using a MettlerDL25 pH meter (MettlerDL25; Mettler–Toledo International Inc.). The NaCl salt content was monitored following the method proposed by Benítez-Cabello et al. (2020). Microbiological analyses were performed on both brine and olive samples after 0, 15, 30, 60, and 80 days of fermentation following the method reported by Pino et al. (2018a,b, 2019). In detail, the drupes were carefully separated from the brine samples, drained, pitted (25 g), transferred into a sterile stomacher bag, 10-fold diluted with Ringer’s solution (Sigma–Aldrich, Milan, Italy), and homogenized for 2–5 min in a stomacher (Lab-Blender, Seward, London). Both brine (10 mL) and olive samples, homogenized as reported above, were serially diluted, using sterile quarter-strength Ringer’s solution, and plated on the following agar media and conditions: plate count agar, incubated at 32°C ± 2°C for 48 h, for total mesophilic bacteria; de Man-Rogosa-Sharp agar, supplemented with cycloheximide (5 mL/L), anaerobically incubated at 32°C for 24–48 h, for LAB count; Sabouraud dextrose agar, supplemented with chloramphenicol (0.05 g/L), incubated at 25°C for 4 days, for yeast count; violet red bile glucose agar, aerobically incubated at 37°C for 24 h, for Enterobacteriaceae count; mannitol salt agar, incubated at 32°C for 48 h, for staphylococci enumeration; MacConkey incubated at 32°C for 24–48 h for Escherichia coli determination; and sulfite polymyxin sulfadiazine agar, anaerobically incubated at 37°C for 24–48 h, for the detection of sulfite-reducing clostridia, as Clostridium perfringens species. All media were purchased from Liofilchem (Roseto degli Abruzzi, Italy). All analyses were performed in triplicate. Results were expressed as log10 CFU/mL for brine and log10 CFU/g for olive samples.

### 16S rRNA Metagenetics Analysis of Olive Fruits

Olives from all samples, at 15 and 80 days of fermentation, were subjected to total DNA extraction using the Dneasy Mericon Food Kit (Qiagen, Milan, Italy) with some modifications. In detail, 12 g of olive samples were diluted with 30 mL of sterile Ringer’s solution and incubated for 2 h at 37°C under constant shaking. After incubation, samples were homogenized in a stomacher apparatus for 3 min at room temperature. The suspension was collected and centrifuged at 10,000 *g* for 10 min at 20°C, and the pellet was washed twice with 30 mL of phosphate-buffered saline, pH 7.4. The pellet was resuspended in 400 μL of Tris-EDTA buffer (10 mM Tris-HCl, 1 mM EDTA, pH 8.0) 1X, and the suspension was transferred into tube containing 0.3 g of zircon beads, added with 150 μL of phenol solution, and homogenized with Precellys Evolution Homogenizer (Bertin Technologies) at 10,000 rpm for 5 min. The obtained suspension was subjected to DNA extraction, following the manufacturer’s instructions. DNA concentration was determined using the fluorimeter Qubit 4.0 (Invitrogen, Carlsbad, CA, United States) before storage at −20°C until use.

Partial 16S rRNA gene sequences were amplified from extracted DNA using the primer pair Probio_Uni and Probio_Rev, targeting the V3 region of the 16S rRNA gene sequence (Milani et al., 2013). 16S rRNA gene amplification and amplicon checks were carried out as previously described (Milani et al., 2013), and 16S rRNA gene sequencing was performed using a MiSeq (Illumina) according to Milani et al. (2013).

### Volatile Organic Compound Analysis by Gas Chromatography–Mass Spectrometry

The VOC profile of all samples at 15 and 80 days of fermentation was investigated by GC–MS. In detail, olives (approximately 100 g), from three different replicates, were pitted and homogenized, and 3.3 g of pulp was placed in a 20-mL glass vial. After the addition of 10 mL NaCl solution (300 g/L) and 10 μL of 2-methyl-4-pentanol (final concentration 75 μg/g) as internal standard, the vial was closed, and extraction by HS-SPME was performed with subsequent analysis by GC–MS, according to the method reported by de Castro et al. (2019). Compound identification was based on mass spectra matching with the standard NIST 08 MS library, on the comparison of retention indices sourced from the NIST Standard Reference Database and from authentic reference standards when available. All analyses were made in triplicate. A PAL COMBI-xt autosampler (CTC combiPAL; CTC Analysis AG, Zwingen, Switzerland) was used to standardize the extraction procedure. The olive samples were kept at 60°C for 15 min. The divinylbenzene/carboxen/polydimethylsiloxane (Supelco, Bellefonte, United States) fiber was exposed to the sample headspace for 60 min (Sánchez et al., 2018). VOC injection was made under splitless mode into the port at 230°C, equipped with a Merlin Microseal. A Clarus 680 (PerkinElmer, Beaconsfield United Kingdom) GC equipped with an Rtx-WAX column (30 m × 0.25-mm internal diameter, 0.25-μm film thickness) (Restek Superchrom, Milano, Italy) was used to thermally desorb and to separate the headspace VOCs. The column temperature was set initially at 35°C for 8 min and then increased to 230°C at 4°C/min and held for 15 min (Montemurro et al., 2020). Helium was used as carrier gas at flow rate of 1 mL/min. A single-quadrupole mass spectrometer Clarus SQ8MS (PerkinElmer) was used to detect the different compounds; the source and transfer line temperatures were 250 and 230°C, respectively. The MS detector system operated in scan mode with mass-to-charge ratio interval 30–350 Da.

### Statistical Analyses

One-way analysis of variance (ANOVA) followed by Tukey *post hoc* multiple-comparisons test was applied to the pH values and microbiological and VOC data from three biological replicates. Differences were considered statistically significant at *p* < 0.05. The differences among fermentation processes of the detected genera were assessed with STAMP software (Parks et al., 2014) using a two-sided Welch *t*-test with a Benjamini–Hochberg false discovery rate correction (*p* < 0.05). In order to correlate the experimental and control samples with the volatile compounds, the data obtained at 15 and 80 days of fermentation were subjected to principal component analysis (PCA). Similarities between the volatile profiles of the inoculated and control olive samples were carried out using the PermutMatrix software. The permutation analysis of the significantly different VOCs was evaluated in the drupe samples with (O1–O2) and without (C5–C8) addition of starter during the fermentation process (T15–T80). All statistical analyses were performed using STATISTICA software (version 7.0 for Windows; TIBCO Software, Palo Alto, CA, United States). In addition, Principal coordinate analysis (PCoA) was performed on the UniFrac distance matrices to show the differences among samples. Correlation analysis between cell density in different microbial groups and VOCs evaluated at 15 and 80 days of fermentation was performed. The Spearman rank correlation was computed in R by using the cor test package^1^ and plotted by using the corrplot package [Wei and Simko (2017); R package ‘‘corrplot’’: Visualization of a Correlation Matrix (version 0.90)].^2^ Results for genera showing significant correlation (*p* < 0.05) were visualized as a correlation matrix (R package “corrplot,” version 0.90) (see text footnote 2).

### Bioinformatics Analysis

FastQ files were processed using a custom script based on the QIIME2 software suite (Caporaso et al., 2010). Paired-end read pairs were assembled to reconstruct the complete Probio_Uni/Probio_Rev amplicons. Quality control retained sequences with a length between 140 and 400 base pairs (bp) and mean sequence quality score > 20, whereas sequences with homopolymers > 7 bp and mismatched primers were omitted. In order to calculate downstream diversity measures (alpha and beta diversity indices, UniFrac analysis), 16S rRNA Amplicon sequence variants (ASVs) were defined at 100% sequence homology using DADA2 (Callahan et al., 2016); ASVs not encompassing at least two sequences of the same sample were removed. Notably, this approach allows highly distinctive taxonomic classification at single-nucleotide accuracy (Callahan et al., 2016). All reads were classified to the lowest possible taxonomic rank using QIIME2 (Caporaso et al., 2010; Bokulich et al., 2018) against SILVA database release 138 (Quast et al., 2013).

Biodiversity within a given sample (alpha diversity) was calculated with Shannon and Chao1 in QIIME2.

Similarities between samples (beta diversity) were calculated by weighted UniFrac (Lozupone and Knight, 2005). The range of similarities is calculated between values 0 and 1. PCoA representations of beta diversity were performed using QIIME2 (Caporaso et al., 2010; Bokulich et al., 2018).

## Results

### Physicochemical Analyses

Table 1 shows pH values of O1, O2, C5, and C8 brine samples measured during the fermentation process. At the beginning of the fermentation, significant differences were found between the C5 and the C8 samples, with values of 6.04 and 6.24, respectively, and the inoculated samples (O1 and O2) exhibited a value of approximately 6.14. As expected, at the beginning of the fermentation, the pH value decreased significantly, reaching a value of approximately 4.5 in the inoculated samples after 24 days, whereas in the controls after 60 days of fermentation. With the exception of the control at 5% of NaCl (C5), a pH < 4.3 was detected at the end of fermentation in all samples, falling within the recommended critical threshold, which would guarantee the microbiological safety of the final product.

**TABLE 1:** Results of pH values in olive brines expressed as means and standard deviations at different times of fermentation.

	Days of fermentation						
Samples	T0	T7	T15	T24	T30	T60	T80
**pH**							
O1	6.17 ± 0.08^aA^	5.74 ± 0.05^bB^	4.85 ± 0.05^cC^	4.50 ± 0.1^bD^	4.42 ± 0.06^cD^	4.33 ± 0.07^bDE^	4.23 ± 0.07^bcE^
O2	6.11 ± 0.05^abA^	5.07 ± 0.08^cB^	4.82 ± 0.08^bC^	4.49 ± 0.1^bD^	4.40 ± 0.10^cD^	4.14 ± 0.06^cE^	4.10 ± 0.06^cE^
C5	6.04 ± 0.10^bA^	5.91 ± 0.05^aAB^	5.70 ± 0.09^aB^	5.43 ± 0.06^aC^	5.40 ± 0.09^aC^	5.04 ± 0.07^aD^	5.04 ± 0.09^aD^
C8	6.24 ± 0.06^aA^	5.66 ± 0.06^bB^	5.61 ± 0.08^aB^	4.70 ± 0.09^bC^	4.70 ± 0.07^bcC^	4.41 ± 0.08^bD^	4.36 ± 0.06^bD^

*O1, fermentation at 5% of NaCl, with the addition of L. plantarum F1.16 and F3.5 strains; O2, fermentation at 5% of NaCl, with the addition of L. plantarum C11C8, F1.16 and F3.5 strains; C5, spontaneous fermentation at 5% of NaCl; C8, spontaneous fermentation at 8% of NaCl.*

*^a–c^Different letters within the same column indicate significant differences at p < 0.05.*

*^A–E^Different letters within the same row indicate significant differences at p < 0.05.*

### Microbiological Data

Table 2 and Supplementary Table 1 show microbial counts detected both in olives and brines, respectively, after 0, 15, 30, 60, and 80 days of fermentation. Overall, in all olive samples (Table 2), a significant decrease in Enterobacteriaceae counts was observed starting from the 30th day of fermentation, in inoculated samples, especially in O2 sample. Similar trend was revealed by coagulase negative staphylococci, whereas coagulase-positive staphylococci counts were below the threshold limit of detection (<1 absent in 25 g) starting from the 15th day of fermentation, in both inoculated samples (Table 2). Viable mesophilic bacteria exhibited different count levels among samples up to 30 days of fermentation, whereas at the 60th day of fermentation, the inoculated samples registered count values of 4.5, with the O2 sample reaching the value of 4.3 log CFU/g, at the end of fermentation. Higher mesophilic bacteria counts were detected in control samples, which showed, at the 60th and 80th days of fermentation, mean values of 6.20 and 5.5 log CFU/g, respectively. Regarding LAB count, no significant difference was found among samples at the beginning of fermentation, whereas a decrease and an increase of approximately 1 log unit, in control samples and in inoculated samples, respectively, were observed at the end of fermentation. Regarding the yeast population, a significant increase (approximately 6 log units) was observed at the 15th day of fermentation, in all samples, especially in C8 samples, reaching a value of 8 log CFU/g. Starting from the 60th day of fermentation, the yeast population decreased, reaching mean values of 3.9 and 5.2 CFU/g in inoculated and control samples, respectively. Furthermore, the presence of sulfite-reducing species and *E. coli* was never detected in any samples at any times.

**TABLE 2:** Microbial counts expressed as log10 CFU/g of 3 replicates ± standard deviation of the main microbial groups detected in O1, O2, C5, and C8 drupe samples during the fermentation.

	Days of fermentation				
Microbial groups	T0	T15	T30	T60	T80
**Enterobacteriaceae**					
O1	4.14 ± 0.06^cA^	3.41 ± 0.10^bB^	2.10 ± 0.16^bC^	<1	<1
O2	3.35 ± 0.10^dA^	2.61 ± 0.07^cB^	<1	<1	<1
C5	4.83 ± 0.08^aA^	4.63 ± 0.06^aA^	3.56 ± 0.10^aB^	2.43 ± 0.12^C^	<1
C8	4.53 ± 0.06^bA^	4.78 ± 0.09^aB^	3.38 ± 0.09^aC^	1.89 ± 0.05^D^	<1
**LAB**					
O1	6.50 ± 0.06^aC^	7.59 ± 0.09^bA^	7.37 ± 0.08^aB^	7.33 ± 0.05^bB^	7.62 ± 0.11^bA^
O2	6.45 ± 0.08^aC^	7.91 ± 0.08^aA^	7.33 ± 0.07^aB^	7.65 ± 0.05^aB^	7.85 ± 0.07^aA^
C5	6.32 ± 0.14^aA^	6.30 ± 0.08^cA^	6.14 ± 0.09^bA^	5.65 ± 0.05^cB^	5.19 ± 0.09^cC^
C8	6.40 ± 0.15^aA^	5.78 ± 0.09^dB^	5.61 ± 0.09^bB^	5.70 ± 0.06^cB^	5.28 ± 0.08^cB^
**Yeasts**					
O1	2.32 ± 0.08^bD^	7.52 ± 0.07^bA^	7.37 ± 0.08^cA^	5.70 ± 0.10^cB^	4.00 ± 0.07^cC^
O2	2.90 ± 0.10^aE^	6.85 ± 0.05^dA^	6.41 ± 0.08^dB^	5.23 ± 0.07^dC^	3.85 ± 0.07^cD^
C5	2.48 ± 0.07^bE^	7.10 ± 0.08^cB^	7.75 ± 0.05^bA^	6.79 ± 0.07^aC^	5.58 ± 0.14^aD^
C8	2.33 ± 0.07^bD^	8.08 ± 0.11^aA^	7.96 ± 0.09^aA^	6.08 ± 0.07^bB^	5.00 ± 0.09^bC^
**Mesophilic Bacteria**					
O1	5.48 ± 0.08^cA^	5.48 ± 0.10^cA^	5.19 ± 0.09^cB^	4.51 ± 0.08^bC^	4.52 ± 0.07^bC^
O2	5.70 ± 0.09^bA^	4.36 ± 0.07^dBC^	4.23 ± 0.06^dC^	4.49 ± 0.08^bB^	4.35 ± 0.09^bBC^
C5	6.30 ± 0.09^aB^	6.85 ± 0.06^bA^	6.81 ± 0.08^bA^	6.18 ± 0.08^aB^	5.78 ± 0.08^aC^
C8	5.00 ± 0.08^dD^	8.08 ± 0.09^aA^	8.26 ± 0.07^aA^	6.30 ± 0.09^aB^	5.30 ± 0.09^aC^
**Coagulase positive staphylococci**					
O1	2.31 ± 0.07^c^	<1	<1	<1	<1
O2	2.11 ± 0.21^c^	<1	<1	<1	<1
C5	3.59 ± 0.09^aB^	4.91 ± 0.08^A^	4.95 ± 0.05^A^	3.70 ± 0.10^B^	<1
C8	3.23 ± 0.11^bB^	4.90 ± 0.08^A^	4.70 ± 0.08^A^	3.33 ± 0.05^B^	<1
**Coagulase negative staphylococci**					
O1	2.85 ± 0.06^cB^	4.20 ± 0.09^aA^	<1	<1	<1
O2	2.63 ± 0.07^bA^	2.30 ± 0.09^cA^	<1	<1	<1
C5	2.91 ± 0.06^aC^	3.33 ± 0.06^bB^	4.70 ± 0.08^aA^	2.60 ± 0.09^C^	<1
C8	2.45 ± 0.09^aA^	1.54 ± 0.07^dB^	2.55 ± 0.06^bA^	<1	<1

*O1, fermentation at 5% of NaCl, with the addition of L. plantarum F1.16 and F3.5 strains; O2, fermentation at 5% of NaCl, with the addition of L. plantarum C11C8, F1.16 and F3.5 strains; C5, spontaneous fermentation at 5% of NaCl; C8, spontaneous fermentation at 8% of NaCl.*

*^a–d^Different letters within the same column indicate significant differences at p < 0.05.*

*^A–E^Different letters within the same row indicate significant differences at p < 0.05.*

Regarding microbiological analyses of brine samples, at the same sampling times mentioned previously, results are reported in the Supplementary Table 1. Enterobacteriaceae counts showed an initial mean value of 3.6 log CFU/mL, with a significant decrease to a mean value of 2.6 log CFU/mL at the 30th day of fermentation. From an initial value of approximately 5.0 log CFU/mL, coagulase-negative staphylococci decreased significantly through the fermentation, reaching a value below the threshold limit of detection (<1 absent in 25 g) from the 60th day of fermentation in inoculated samples. With the exception of C5 sample, low values of coagulase-positive staphylococci were detected in all samples at the beginning of fermentation, which decreased significantly starting from the 30th day. Mesophilic bacteria decreased through the fermentation in all samples, reaching a mean final value of 6.1 log CFU/mL. LAB counts were higher in inoculated samples than in controls, showing a constant trend up to the end of fermentation. Yeast population showed a reduction of almost 3 log units, with the exception of inoculated sample O1, where a 2 log units’ decrease was observed. The presence of *E. coli* was never detected in sample O2; its complete inhibition was revealed in O1 and C8 samples starting from the 30th day and from the 60th day in C5 sample. Sulfite-reducing species were not detected (absent in 25 g) in any tested samples.

### Taxonomy Analysis of Table-Olives Microbiota

**FIGURE 2:**
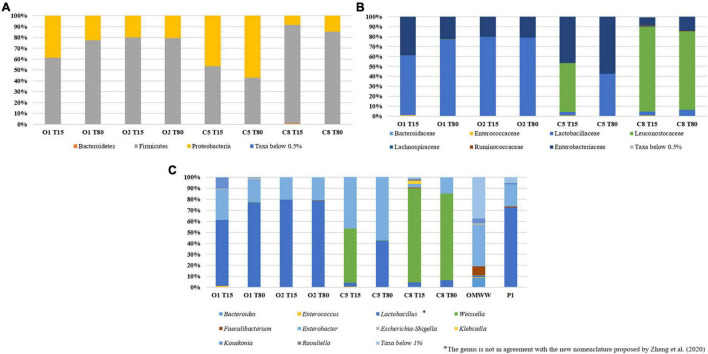
Relative abundance (%) of bacterial phyla **(A)**, family **(B)**, and genera **(C)** found on O1, O2, C5, and C8 olive samples at 15 and 80 days of fermentation.

**FIGURE 3:**
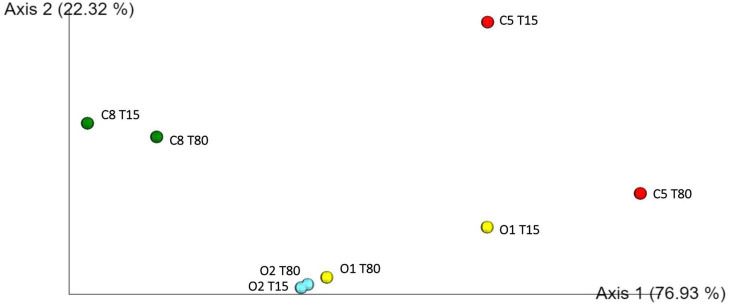
Principal coordinate analysis (PCoA) plot of 16S sequences.

Inoculated and control table-olives samples both at 15 and 80 days of fermentation were subjected to sequencing of the V3 region of the 16S rRNA gene. Per sample number of reads, denoised reads, Shannon and Chao 1 indices, are reported in Supplementary Table 2. Overall, the analysis allowed obtaining a total of 449,654 bacterial sequences with an average of 56,207 sequences for each analyzed sample. The Chao1 index increased from 15 to 80 days of fermentation except in the control sample C8, in which a decrease in Chao1 index was observed during the fermentation process (Supplementary Table 2). A satisfactory coverage of the bacterial diversity was found for all the analyzed samples with Good’s coverage values greater than 99% (Supplementary Table 2), which was confirmed by rarefaction curves analysis (Supplementary Figure 1, only Richness data are shown). The bacterial biota of the analyzed olive samples was covered by 3 phyla (Figure 2A), 7 families (Figure 2B), and 10 genera (Figure 2C) (relative abundance > 0.5%). In detail, Firmicutes and Proteobacteria were the phyla mainly detected. Among these, Firmicutes showed the highest relative abundance in all samples, with the exception of C5 sample at 80 days of fermentation. The Bacteroidetes phylum was found, even at low abundance, only in C8 sample at the beginning of fermentation (Figure 2A). Zooming on the microbiota profile at family level (Figure 2B), Lactobacillaceae family was mainly detected in inoculated samples at both 15 and 80 days of fermentation, whereas Leuconostocaceae family was revealed in control samples. Enterobacteriaceae family showed the highest occurrence in C5 sample at 80 days of fermentation (Figure 2B). The prevalence of 10 genera (relative abundance > 0.5%) within table-olives samples is depicted in Figure 2C. At the genus level (Figure 2C), *Lactobacillus* (according with the old nomenclature) dominated the O1 and O2 olive samples at both 15 and 80 days of fermentation. *Weissella* was mainly detected in C8 sample both at the beginning of fermentation (85.09%) and at the end of the process (78.34%), and in C5 sample at 15 days of fermentation (49.05%). High occurrence of *Enterobacter* genus was revealed only in C5 sample. The genera *Bacteroides*, *Faecalibacterium*, *Klebsiella*, and *Raoultella* were detected only in C8 sample at 15 days of fermentation (Figure 2C). By comparing the different fermentation processes, between inoculated and control samples, with a two-sided Welch test, genera *Enterobacter*, *Lactobacillus*, and *Weissella* resulted to be statistically different (Supplementary Figure 2). Looking at group statistics, *Enterobacter* was statistically higher (*p* = 0.030) in C5 when compared with C8. The control samples with NaCl 8% were proven to contain significantly higher levels of *Weissella* (compared with O1 and O2), whereas a significantly lower amount of *Lactobacillus* was found when compared with O2. Figure 3 shows the unweighted UniFrac analysis based in PCoA of 16S sequences. The PCoA allowed to group samples based on both treatment and fermentation time along the Axes 1 (PC1) that explained more than 75% of total variance. High similarity among C8 olive samples at 15 and 80 days of fermentation was recorded. In addition, based on the PCoA, olive samples O2, at both 15 and 80 days of fermentation, and olive sample O1 at 80 days of fermentation grouped together.

### Evaluation of Volatile Organic Compounds in Olive Samples

**FIGURE 4:**
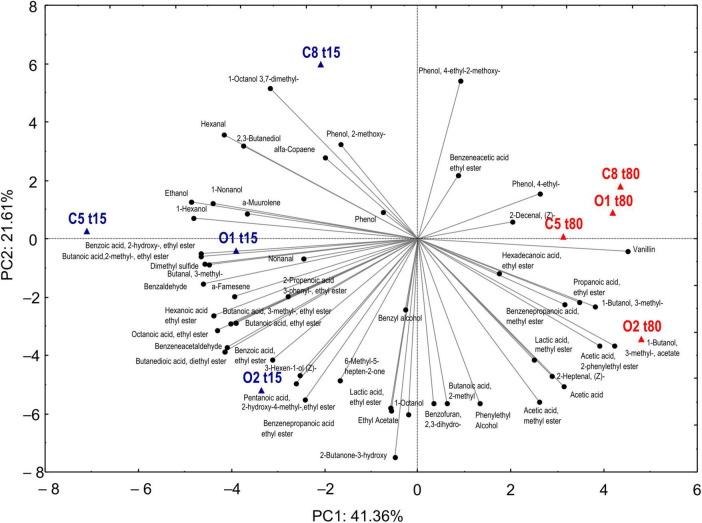
Principal component analysis of VOCS evaluated in drupe samples with (O1–O2) and without (C5–C8) addition of starters during fermentation (T15–T80).

**FIGURE 5:**
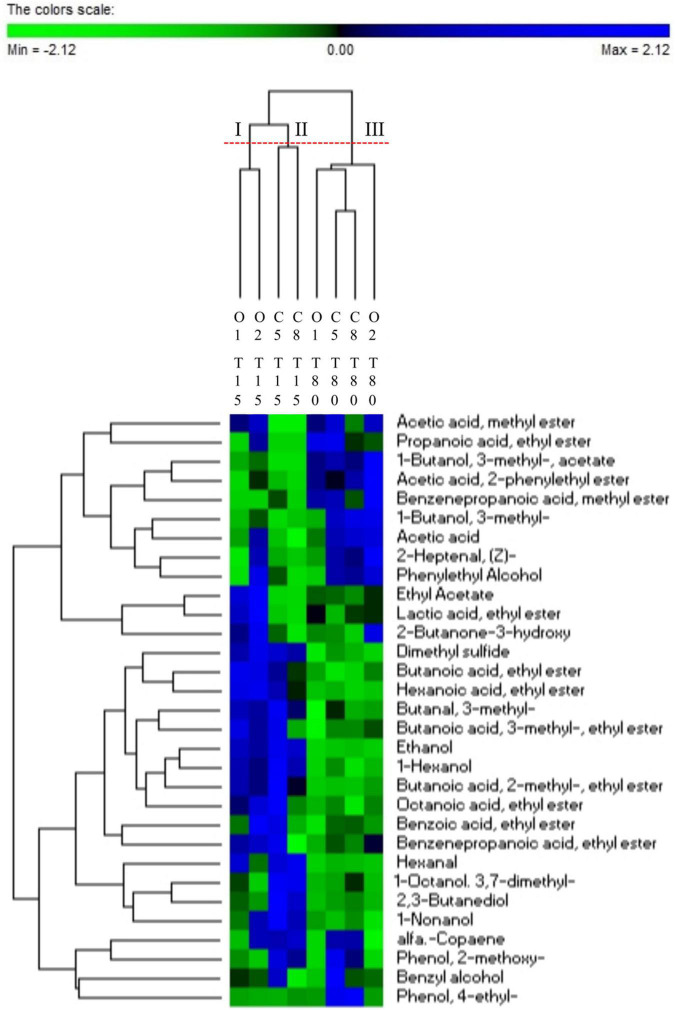
Permutation analysis of significantly different VOCs (ANOVA test corrected with Tukey) evaluated in drupe samples with (O1–O2) and without (C5–C8) addition of starters during fermentation (T15–T80).

An objective comparison of volatile metabolic profile in table-olives samples at 15 and 80 days of fermentation was performed based on qualitative and quantitative differences in VOCs using HS-SPME GC–MS methodology. Fifty-two volatile compounds were identified and grouped according to chemical classes, that is, alcohols (9), esters (21), aldehydes (7), phenols (5), ketones (2), organic acids (2), terpenes (3), and others (3). The two principal factors of PCA analysis (PC1 and PC2), explaining 63% of the total variance, showed that drupes were clearly distributed according to the time of fermentation (Figure 4). Thirty-one of 52 volatile compounds showed a significant difference among the uninoculated and inoculated table olives during the fermentation (Supplementary Table 3). The significant different compounds, resulting from ANOVA, were used for the permutation analysis (Figure 5). The permutation analysis clearly showed that table olives were grouped into three clusters according to the fermentation process and time. Indeed, cluster I grouped all inoculated table olives at 15 days, cluster II included the uninoculated samples at the same time, whereas cluster III encompassed samples at 80 days of fermentation. It should be noted that O2 sample at 15 days of fermentation was characterized by the highest content of 2-butanone-3-hydroxy (acetoin), ethyl acetate, and lactic acid ethyl ester. The same sample after 80 days of fermentation was characterized by the highest content of acetic acid, 3-methyl-1-butanol and its derivatives esters as well as phenylethyl alcohol, acetic acid 2-methyl ester, 2-heptanal, and benzene propanoic acid methyl ester. It is interesting to note that salt content did not discriminate the VOC profile between control samples at the end of fermentation. In detail, compared with inoculated samples (O1 and O2) at 80 days of fermentation, C5 and C8 were characterized by the higher content (*p* < 0.05) of 4-ethyl-phenol and the 2-methoxy-phenol. Focusing on alcohols, it is possible to assert that after 80 days of fermentation, a significant decrease in this class of compounds, except for phenylethyl alcohol, 3-methyl-1-butanol, and benzyl alcohol, was observed. A similar trend was detected for aldehydes, with butanal-3-methyl and hexanal showing a significant decrease during the fermentation. An overall increase for acetic acid and derivative esters in all samples was detected during the fermentation. Of note, ethyl acetate decreases in the inoculated olives (O1 and O2) after 80 days of fermentation, whereas an opposite trend was assessed in the uninoculated (C5 and C8) samples.

### Correlation Between Volatile Organic Compound Profile and Viable Microbial Groups

**FIGURE 6:**
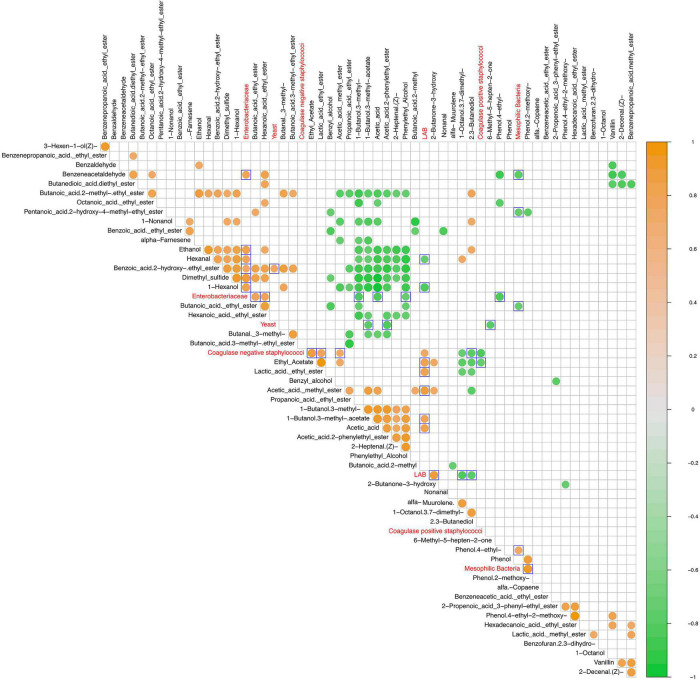
Spearman correlation matrix between VOCs (black font characters) and microbial groups (red font characters) detected values after 15 and 80 days of fermentation in olive drupes. The normalized scaled matrices were merged and used for correlation computing. Only statistically significant correlations (*p* < 0.05) were plotted. The color graduated scale ranges from −1 (green: negative correlations) to 1 (orange: positive correlations). Blue delimited square marked the outgroup comparisons.

Statistically significant correlations between microbial groups and VOCs are shown in Figure 6. LAB cell density values positively correlated with acetic acid (*p* = 0.017) and its derivatives ethyl and methyl esters (acetic acid methyl ester, ethyl acetate, 1-butanol 3-methyl-acetate), as well as with lactic acid ethyl ester (*p* = 0.009) and 2-butanone-3-hydroxy (acetoin) (*p* = 0.006). Looking at mesophilic bacteria group, a positive correlation was detected with phenol-2-methoxy and phenol-4-ethyl, which was negatively correlated with the Enterobacteriaceae group (*p* = 0.011). Moreover, the latter microbial group showed a positive correlation with alcohols (ethanol, 1-hexanol), esters (butanoic acid ethyl ester, hexanoic acid ethyl ester, benzoic acid 2-hydroxy ethyl ester), aldehydes (hexanal and benzeneacetaldehyde), and dimethyl sulfide and a negative correlation with acetic acid (*p* = 0.012), 1-butanol 3-methyl- (*p* = 0.015), and phenylethyl alcohol (*p* = 0.039). Regarding yeasts, a positive correlation with benzoic acid 2-hydroxy-ethyl ester was revealed, whereas 1-butanol-3-methyl-acetate, acetic acid-2-phenylethyl ester, and 6-methyl-5-hepten-2-one were negatively correlated. The above detected remarks were partially confirmed by the correlation analysis between relative abundances of bacterial genera and VOCs (Supplementary Figure 3). In detail, *Lactobacillus* was positively correlated (*p* < 0.05) with derivative esters of acetic and lactic acid (ethyl acetate, acetic acid methyl ester, and lactic acid ethyl ester) and 2-butanone-3-hydroxy. On the other hand, this group was significantly negatively correlated with 2–3 butanediol, 1-octanol 3,7-dimethyl-, muurolene, and phenol-2-methoxy. An opposite trend was observed for *Weissella*, showing a negative correlation (*p* < 0.05) with ethyl acetate, acetic acid methyl ester, butanoic acid-2-methyl, and 2-butanone-3-hydroxy, whereas positive correlations were found with 1-octanol 3,7- dimethyl-, muurolene, and phenol, 4-ethyl-2-methoxy. *Enterobacter* was negatively correlated (*p* < 0.05) with 2.3-dihydro-benzofuran.

## Discussion

Table olives are an integral part of the Mediterranean diet. Recently, several studies highlighted that the use of selected starter cultures allows reducing the debittering time and the risk of survival/growth of spoilage or pathogenic microorganisms (Panagou et al., 2008; Bevilacqua et al., 2013; Bonatsou et al., 2015; Pino et al., 2019). Studies conducted on table olive highlighted the importance of selection strains with β-glucosidase activity and able to grow at low salt content as promising strategy to produce safe and healthy products (Tataridou and Kotzekidou, 2015; Pino et al., 2018b,^2019^; Anagnostopoulos et al., 2020; Perpetuini et al., 2020), in accordance with the World Health Organization recommendations. In this contest, understanding the composition, diversity, and functioning of microbial ecosystems was the relevant challenge of the present study. In detail, the effect of two different starter cultures obtained using three selected β-glucosidase–positive *L. plantarum* strains (C11C8, F1.16, and F3.5) was investigated on microbial composition and on VOC of Sicilian table olives, processed under 5 and 8% of salt, during fermentation up to 80 days. Our results revealed that inoculated table olives exhibited a more pronounced drop in pH, reaching values ≤ 4.5 starting from the 15th day, indicating a faster brine acidification in respect to control samples. The data confirm the importance of starter cultures for ensuring the microbiological safety of final product, in accordance to previous studies (Corsetti et al., 2012; Martorana et al., 2017; Pino et al., 2019). When spontaneous fermentation was conducted, the salt content exerts a selective pressure on microbial composition. Accordingly, metagenetics data revealed an abundance of *Enterobacter* species (57%) and a dominance of *Weissella* species (78%) in control samples at 5 and 8% of NaCl, respectively. The *Weissella* genus was the most abundant detected genus, according to results reported by Lucena-Padrós et al. (2014), who described this genus in treated Manzanilla table olives. *Enterobacter* often found in fresh fruits and associated with Spanish-style and with spontaneous fermentation table olives (Cocolin et al., 2013; De Angelis et al., 2015) was drastically reduced in inoculated samples, whereas it persisted mainly in control samples processed at lower salt content. This finding is not in line with our microbiological data, which highlighted the complete inhibition of Enterobacteriaceae in final product. This incongruity could be explained by the fact that culture-independent techniques applied on total bacteria could not distinguish truly active from dead or compromised microbial cells (Kazou et al., 2020), supporting the importance to apply an integrated approach, coupling culture-dependent and independent methods, for such a complex microbial ecosystem. Based on the dual approach, *Lactobacillus* genus was found dominating the fermentation of samples inoculated with *L. plantarum* strains as confirmed by the comparative analysis among different fermentation processes. The use of *L. plantarum* strains could also influence the table-olives ecosystem by preventing the spoilage microbial growth and positively contributing to pleasant VOCs formation in the final product, as previously reported (Perpetuini et al., 2020). This was also in line with our findings showing a positive correlation of *Lactobacillus* with esters of acetic and lactic acid as well as acetoin. The presence of high metabolically active lactobacilli on the drupes confirms the ability of *L. plantarum* to adhere and colonize the fruit surface, according to previously reported data (Lavermicocca et al., 2005; Arroyo-López et al., 2012; Domínguez-Manzano et al., 2012; Blana et al., 2014; Benítez-Cabello et al., 2015; De Angelis et al., 2015; Grounta et al., 2015, 2016; Faten et al., 2016; Perpetuini et al., 2016; Montoro et al., 2018). According to De Angelis et al. (2015) and to Anagnostopoulos et al. (2020), in the present study the clear dominance of *L. plantarum* in inoculated samples confirms that the used strains were able to withstand the competition with microorganisms naturally present in the drupes and to persist up to the end of fermentation (Hurtado et al., 2012; Randazzo et al., 2014; Benítez-Cabello et al., 2020). Both coagulase-positive and -negative staphylococci were strongly reduced from the 30th day of fermentation both in experimental olive and brine samples. In addition, *Kosakonia* genus, frequently found in environmental sources, such as soil, plants, and trees, enclosing nitrogen-fixing bacteria (Brady et al., 2013), was revealed (9.9%) only in inoculated sample at the 15th day of fermentation, disappearing at the end of the process. Gram-negative bacteria, such as *Raoultella* genus, frequently recovered from water, soil, and plants, were detected in uninoculated samples, corroborating its presence in naturally fermented table olives, as reported by other authors (Ercolini et al., 2006; Penland et al., 2020). The detection of this bacterial genus in olives is not uncommon. Indeed, Maza-Márquez et al. (2017) revealed the presence of species belonging to *Raoultella* genus, capable of degrading phenolic compounds in olive wastewater.

In the present study, the volatilomics approach revealed the presence of 52 VOCs, including alcohols, esters, aldehydes, phenols, ketones, organic acid, and terpenes, typically found in table olives (Vaccalluzzo et al., 2020a). According to Randazzo et al. (2014) and Pino et al. (2018b,2019), our data disclosed that the use of lactobacilli culture affects the profile of Sicilian table olives in terms of VOCs abundances. Among VOCs, ester compounds were the most abundant, especially in inoculated samples, in particular, acetate esters, which are synthesized by an alcohol-acyl-transferase that catalyzes the esterification of volatile alcohols with acetyl CoA molecules to produce volatile esters and free CoAeSH (Salas, 2004). The 3-methyl-1-butanol acetate and the 2-methyl ester compounds, mainly detected in inoculated samples, have been reported as responsible of pleasant flavors (Sabatini and Marsilio, 2008). The occurrence of off-flavor may be attributed to the formation of malodorous compounds, such as 4-ethylphenol (Brenes et al., 2004). This phenolic compound is generally formed by microorganisms via the decarboxylation of *p*-coumaric acid to form 4- vinylphenol and reduction of the latter compound (Dias et al., 2003; Rodríguez et al., 2009). One of the most outstanding differences between inoculated and uninoculated samples was assessed for the 4-ethylphenol concentration. The highest concentration of this phenolic compound was detected in control samples at 80 days of fermentation. Although, the 4-ethylphenol concentration is related to storage time (Manthos et al., 2021), in the present study the addition of starter cultures seemed to inhibit its production as off-flavor. It is also interesting to point out that butyric, propionic, and cyclohexanoic acids, which are responsible for zapatera off-odors (de Castro et al., 2018), were never found in tested samples, confirming the inhibition of *Clostridium* and *Propionibacterium* genera by selected starter cultures. From a qualitative perspective, no major differences were observed regarding VOC profiles among different fermentation processes, so the use of starter does not appear to favor any specific aroma compound. According to Penland et al. (2020), VOC profile may probably be related mainly to cultivar rather than changes in the microbial community during the fermentation process.

## Conclusion

The present study confirmed that a dual approach based on culture-dependent, metagenetic, and volatilomic techniques allowed exploring in depth the microbial composition and related VOCs of Sicilian table olives. Metagenetics revealed the dominance of *Lactobacillus* genus in inoculated samples, whereas spoilage bacteria were detected only in control samples. *L. plantarum* strains with oleuropein-degrading activity, used as tailored starter culture, were able to drive the fermentation process at low salt content, ensuring the microbiological safety and the pleasant flavors of the final product.

## Data Availability Statement

The original contributions presented in the study are included in the article/Supplementary Material, further inquiries can be directed to the corresponding author/s. The raw reads for 16S rRNA Sequencing were deposited into the NCBI Sequence Read Archive database (accession: PRJNA 675996).

## Author Contributions

AV and GC performed the experiments, analyzed the data, wrote the manuscript, and designed the study. CC and CR obtained the funding and supervised the study. AP, CC, CR, and FMC edited and proofread the manuscript. AV, GC, and PF performed the experiments and analyzed all the data. FMC performed bioinformatics and statistical analyses. AV and CR drafted the manuscript. AV, GC, AP, and PF contributed to data curation. All authors contributed to the article.

## Conflict of Interest

CR, AP, and CC were employed by the ProBioEtna srl, Catania, Italy. This study received funding from VERIFICO project. The funder was not involved in the study design, collection, analysis, interpretation of data, the writing of this article or the decision to submit it for publication. The remaining authors declare that the research was conducted in the absence of any commercial or financial relationships that could be construed as a potential conflict of interest.

## Publisher’s Note

All claims expressed in this article are solely those of the authors and do not necessarily represent those of their affiliated organizations, or those of the publisher, the editors and the reviewers. Any product that may be evaluated in this article, or claim that may be made by its manufacturer, is not guaranteed or endorsed by the publisher.

## Footnotes

1
https://www.rdocumentation.org/packages/stats/versions/3.6.2/topics/cor

2
https://github.com/taiyun/corrplot
