# Supplementary material for: Metagenetic and Volatilomic Approaches to Elucidate the Effect of Lactiplantibacillus plantarum Starter Cultures on Sicilian Table Olives
Source: Front Microbiol. 2022 Feb 25;12:771636. doi: 10.3389/fmicb.2021.771636 (PMC8914321; doi:10.3389/fmicb.2021.771636)
Supplement: Supplementary file 1 [file Data_Sheet_1.PDF]

Supplementary Material

1 Supplementary Figures and Tables

1.1 Supplementary Figures

Supplementary Figure 1. Richness data of Chao1 index.

Supplementary Figure 2. Significant differences (Welch's t-test corrected with Benjamini-Hochberg) evaluated at genus level in table olives manufactured with different fermentation process. Boxplots indicating genus sample distribution (proportion of sequences) in O1: fermentation at 5% of NaCl, with the addition of *L. plantarum* F1.16 and F3.5 strains; O2: fermentation at 5% of NaCl, with the addition of *L. plantarum* C11C8, F1.16 and F3.5 strains; C5: spontaneous fermentation at 5% of NaCl; C8: spontaneous fermentation at 8% of NaCl.

Supplementary Figure 3. Spearman correlation matrix between VOCs (black font characters) and relative abundances of genera (red font characters) detected after 15 and 80 days of fermentation in olive drupes. The normalized scaled matrices were merged and used for correlation

computing. Only statistically significant correlations ($p<0.05$) were plotted. The colour graduated scale ranges from -1 (green - negative correlations) to 1 (orange - positive correlations). Blue delimited square marked the outgroup comparisons.

1.2 Supplementary Tables

Supplementary Table 1. Microbial counts expressed as log10 CFU/ml of 3 replicates ± standard deviation of the main microbial groups detected in O1, O2, C5 and C8 brine samples during the fermentation process.

Microbial groups	Days of fermentation				
	T0	T15	T30	T60	T80
			Enterobacteriaceae		
O1	3.54±0.06^{aB}	4.49±0.10^{bcA}	2.44±0.06^{bC}	<1	<1
O2	3.53±0.12^{aB}	4.38±0.10^{cA}	2.35±0.05^{bC}	<1	<1
C5	3.72±0.07^{aB}	4.72±0.07^{aA}	2.76±0.10^{aC}	<1	<1
C8	3.63±0.07^{aB}	4.61±0.07^{abA}	2.68±0.10^{aC}	<1	<1
			LAB		
O1	7.83±0.05^{cA}	7.58±0.07^{aB}	7.50±0.09^{aB}	7.23±0.07^{aC}	7.20±0.09^{aC}
O2	8.20±0.07^{aA}	7.58±0.08^{aB}	7.25±0.08^{bC}	7.04±0.17^{aC}	7.26±0.10^{aC}
C5	7.52±0.10^{bA}	7.01±0.16^{bC}	7.30±0.08^{abAB}	7.20±0.08^{aBC}	6.23±0.06^{bD}
C8	7.50±0.09^{cA}	6.34±0.11^{cB}	7.30±0.09^{abA}	6.34±0.06^{bB}	5.78±0.11^{cC}
			Yeasts		
O1	7.40±0.09^{bA}	7.40±0.13^{bA}	7.22±0.06^{bA}	7.21±0.08^{aA}	5.23±0.07^{aB}
O2	7.37±0.08^{bA}	7.36±0.06^{bA}	6.32±0.08^{cC}	6.88±0.07^{bB}	4.85±0.07^{bD}
C5	7.80±0.09^{aA}	7.77±0.13^{aA}	7.40±0.09^{bB}	6.93±0.07^{bC}	4.70±0.09^{bcD}
C8	7.54±0.06^{bAB}	7.45±0.11^{bB}	7.74±0.08^{aA}	7.37±0.08^{aB}	4.61±0.08^{cC}
			Mesophilic Bacteria		
O1	7.50±0.08^{bA}	6.80±0.09^{cB}	6.20±0.09^{dC}	6.11±0.09^{cC}	5.67±0.20^{cD}
O2	7.00±0.10^{cA}	6.78±0.07^{cB}	6.54±0.05^{cC}	6.04±0.06^{cD}	5.77±0.08^{cE}
C5	8.20±0.07^{aA}	8.15±0.08^{bA}	7.30±0.07^{bB}	6.74±0.12^{bC}	6.20±0.16^{bD}
C8	8.33±0.08^{aAB}	8.58±0.08^{aA}	8.08±0.16^{aB}	7.00±0.07^{aC}	6.90±0.09^{aC}
			Coagulase positive staphylococci		

O1	3.70±0.07^{cA}	<1	<1	<1	<1
O2	2.33±0.12^{d}	<1	<1	<1	<1
C5	5.32±0.08^{aB}	6.81±0.08^{aA}	3.90±0.09^{C}	3.48±0.10^{D}	<1
C8	4.30±0.09^{b}	5.95±0.10^{b}	<1	<1	<1
Coagulase negative staphylococci					
O1	4.55±0.09^{cA}	4.50±0.09^{cAB}	2.31±0.08^{cC}	<1	<1
O2	5.00±0.15^{bA}	4.11±0.12^{dB}	2.45±0.06^{cC}	<1	<1
C5	5.70±0.09^{aA}	5.76±0.08^{aA}	6.84±0.11^{aB}	4.44±0.12^{C}	<1
C8	5.10±0.09^{bA}	5.00±0.09^{bA}	4.94±0.10^{bA}	2.95±0.12^{B}	<1
Escherichia coli					
O1	2.10±0.20^{c}	1.91±0.08^{c}	<1	<1	<1
O2	<1	<1	<1	<1	<1
C5	5.71±0.16^{aA}	5.71±0.21^{aA}	3.63±0.06^{B}	<1	<1
C8	4.20±0.10^{b}	4.63±0.06^{b}	<1	<1	<1

Supplementary Table 2. Number of sequences analysed, observed taxa, and biodiversity measures of total 16S rRNA gene of O1, O2, C5, and C8 olives samples at 15 and 80 days of fermentation

Sample	Number of reads	Shannon	Chao1	Good coverage (%)
O1 T15	46554	1.62	22	99.54
O1 T80	73888	1.08	31	99.53
O2 T15	63960	0.98	24.31	99.62
O2 T80	53669	1.03	27.63	99.60
C5 T15	42679	1.75	11.9	99.70
C5 T80	52016	1.38	15.1	99.72
C8 T15	52726	1.59	69.16	99.84
C8 T80	64162	1.52	38.53	99.82

*O1: fermentation at 5% of NaCl, with the addition of *L. plantarum* F1.16 and F3.5 strains; O2: fermentation at 5% of NaCl, with the addition of *L. plantarum* C11C8, F1.16 and F3.5 strains; C5: spontaneous fermentation at 5% of NaCl; C8: spontaneous fermentation at 8% of NaCl.

Supplementary Table 3. Statistically different VOCs of drupe samples, expressed as μg/g, evaluated at different salt concentrations (NaCl 5 and 8 %) under controlled (O1 and O2) and spontaneous (C5 and C8) fermentation after 15 and 80 days.

	O1		O2		C5		C8	
	**T15**	**T80**	**T15**	**T80**	**T15**	**T80**	**T15**	**T80**
Ethanol	248.76±43.66^{a}	56.87±13.95^{b}	219.53±29.30^{a}	66.92±4.28^{b}	317.88±80.53^{a}	75.37±35.84^{b}	260.36±45.05^{a}	84.21±30.38^{b}
1-Butanol, 3-methyl-	29.73±10.25^{bc}	31.57±8.77^{bc}	41.77±9.80^{b}	71±16.75^{a}	22.71±4.21^{c}	61.13±29.22^{ab}	28.31±21.07^{bc}	69.34±24.34^{a}
1-Octanol. 3,7-dimethyl-	12.52±1.66^{ab}	5.63±9.76^{b}	2.95±5.11^{b}	4.88±8.46^{b}	32.03±5.69^{a}	7.33±12.69^{b}	29.81±2.93^{a}	13.12±4.36^{ab}
1-Hexanol	5.47±2.48^{ab}	2.2±0.18^{b}	4.82±0.60^{ab}	2.79±0.78^{ab}	7.58±3.11^{a}	3.41±0.92^{ab}	5.55±2.58^{ab}	1.83±1.58^{b}
3-Hexen-1-ol (Z)-	10.59±18.34^{a}	8.63±8.41^{a}	16.76±2.09^{a}	12.73±16.64^{a}	24.53±11.28^{a}	4.12±7.14^{a}	nd	6.82±7.20^{a}
1-Octanol	4.16±1.77^{a}	2.99±0.43^{a}	4.63±1.69^{a}	5.55±1.39^{a}	4.73±2.38^{a}	5.5±1.48^{a}	1.89±1.67^{a}	2.58±0.74^{a}
1-Nonanol	3.84±3.34^{bc}	3.02±2.64^{bc}	7.86±0.92^{ab}	nd	12.4±3.12^{a}	1.83±3.17^{bc}	7.84±3^{ab}	3.39±3.56^{bc}
Benzyl alcohol	15.49±6.05^{ab}	12.06±2.22^{b}	15.01±0.86^{ab}	14.69±3.84^{ab}	19.43±3.13^{ab}	24.05±5.09^{a}	9.67±1.92^{b}	15.09±4.14^{ab}
Phenylethyl Alcohol	79.8±36.07^{c}	98.78±34.56^{bc}	255.9±116.41^{a}	237.59±43.68^{a}	153.45±61.66^{b}	205.31±84.67^{a}	84.85±18.03^{c}	195.22±77.54^{a}
**Total Alcohols**	**410.36**	**221.75**	**569.23**	**416.15**	**594.74**	**388.05**	**428.28**	**391.6**
Butanal, 3-methyl-	55.5±3.57^{ab}	nd	50.78±10.75^{b}	27.87±7.08^{c}	73.99±12.52^{a}	38.98±7.81^{bc}	44.8±3.17^{bc}	25.3±0.35^{c}
Hexanal	46.07±36.08^{ab}	1.48±0.14^{c}	17.63±7.09^{bc}	4.02±2.33^{c}	47.76±0.63^{ab}	7.61±6.08^{c}	57.86±8.73^{a}	6.4±1.33^{c}
2-Heptenal, (Z)-	0.81±1.41^{b}	4.04±0.99^{ab}	8.8±4.53^{ab}	12.91±4.7^{a}	3.58±3.11^{b}	8.33±3.57^{ab}	2.53±2.33^{b}	7.23±3.34^{ab}
Nonanal	32.69±17.80^{a}	13.52±1.06^{a}	17.44±2.31^{a}	23.1±7.72^{a}	28.15±3.59^{a}	26.48±8.49^{a}	19.66±5.21^{a}	17.63±4.32^{a}
Benzaldehyde	24.73±9.52^{a}	15.44±6.29^{a}	29.62±4.23^{a}	18.86±1.07^{a}	34.87±8.74^{a}	21.61±10.47^{a}	23.64±3.34^{a}	18.44±8.99^{a}
Benzeneacetaldehyde	27.83±10.91^{a}	10.43±0.31^{a}	31.6±13.11^{a}	14.57±5.16^{a}	24.85±5.89^{a}	12.31±4.57^{a}	15.5±4.30^{a}	5.81±5.15^{a}
2-Decenal, (Z)-	nd	0.81±1.40^{a}	nd	5.18±0.70^{a}	3.79±6.57a	3.89±1.20^{a}	2.01±1.90^{a}	4.09±1.28^{a}
**Total Aldehydes**	**187.63**	**45.72**	**155.87**	**106.51**	**216.99**	**119.21**	**166**	**84.9**
Acetic acid, methyl ester	14.86±2.75^{ab}	14.7±4.57^{ab}	19.24±3.46^{a}	18.97±5.06^{a}	nd	20.64±2.05^{a}	nd	9.57±1.43^{b}
Ethyl Acetate	255.42±74.82^{ab}	116.23±20.80^{bc}	351.38±137.06^{a}	130.74±28.3^{bc}	17.87±4.20^{c}	111.17±41.53^{bc}	11.16±3.23^{c}	90.3±45.48^{bc}
Propanoic acid, ethyl ester	nd	7.95±3.99^{a}	5.15±8.92^{ab}	2.92±5.06^{c}	nd	8.27±0.59^{a}	nd	3.3±5.72^{c}
Butanoic acid, ethyl ester	6.2±1.45^{a}	1.6±0.28^{cd}	5.68±1.85^{ab}	2.07±0.38^{cd}	3.87±1.85abc	nd	2.7±0.49^{bcd}	0.62±1.08^{cd}
Butanoic acid, 2-methyl-, ethyl ester	10.2±4.52^{ab}	2.78±0.77^{b}	8.75±3.07^{ab}	4.8±2.46^{b}	17.08±6.53^{a}	3.37±5.84^{b}	7.36±1.79^{ab}	3.24±2.81^{b}
Butanoic acid, 3-methyl-, ethyl ester	11.39±4.44^{a}	2.47±2.31^{c}	9.07±3.13^{ab}	7.18±3.31^{b}	11.87±6.44^{a}	6.43±6.21^{b}	5.61±1.57^{bc}	6.37±2.71^{b}
1-Butanol, 3-methyl-, acetate	7.57±2.93^{cd}	20.68±8.66^{bc}	12.66±5.30^{bcd}	41.49±13.02^{a}	nd	24.62±0.44^{ab}	nd	20.25±2.85^{bc}

Hexanoic acid, ethyl ester	18.24±10.40^{a}	3.43±2.99^{b}	19.78±5.16^{a}	3.24±3.18^{b}	14.02±3.49^{ab}	4.6±1.26^{b}	9.21±1.711^{ab}	2.14±1.95^{b}
Lactic acid, methyl ester	nd	1.59±1.50^{a}	0.9±1.57^{a}	2.73±2.62^{a}	nd	nd	nd	nd
Lactic acid, ethyl ester	62.21±12.10^{ab}	35.67±13.62^{bc}	99.42±40.83^{a}	34.34±5.06^{bc}	7.21±8.66^{c}	12.11±17.88^{c}	nd	33.34±6.68^{bc}
Octanoic acid, ethyl ester	6.29±1.99^{abc}	2.8±1.98^{bc}	9.48±2.54^{ab}	4.11±1.95^{bc}	12.08±3.43^{a}	4.02±3.61^{bc}	4.03±4.12^{bc}	0.4±0.70^{c}
Pentanoic acid, 2-hydroxy-4-methyl-, ethyl ester	3.52±3.62^{a}	3±0.92^{a}	6.39±3.24^{a}	1.74±1.51^{a}	3.8±1.3^{a}	nd	0.59±1.02^{a}	1.71±1.49^{a}
Benzoic acid, ethyl ester	5.88±2.69^{ab}	4.06±2.00^{b}	9.88±1.40^{a}	5.48±1.18^{ab}	8.74±1.77^{ab}	6.01±2.99^{ab}	5.14±1.37^{ab}	6.06±0.71^{ab}
Butanedioic acid, diethyl ester	19±8.20^{a}	7.26±8.40^{a}	21.83±7.28^{a}	4.4±2.43^{a}	22.7±25.98^{a}	1.26±2.19^{a}	2.63±2.38^{a}	2.24±2.65^{a}
Benzeneacetic acid, ethyl ester	4.21±3.84^{a}	3.22±2.54^{a}	1.83±1.60^{a}	2.1±1.97^{a}	3.11±2.87^{a}	3.67±3.18^{a}	2.13±1.92^{a}	4.02±2.08^{a}
Benzoic acid, 2-hydroxy-, ethyl ester	6.84±2.35^{a}	1.38±1.25^{a}	4.04±1.26^{a}	1.67±1.52^{a}	6.59±4.32^{a}	2.5±2.43^{a}	3.57±1.09^{a}	0.85±1.47^{a}
Acetic acid, 2-phenylethyl ester	nd	5.01±2.18^{ab}	3.59±3.81^{b}	10.48±0.64^{a}	1.12±1.93^{b}	3.77±3.27^{b}	nd	5.72±2.92^{ab}
Benzenepropanoic acid, methyl ester	nd	1.41±0.35^{ab}	nd	2.61±0.96^{a}	0.78±1.35^{ab}	1.54±1.42^{ab}	nd	0.76±1.31^{ab}
Benzenepropanoic acid, ethyl ester	11.52±3.74^{a}	6.76±2.71^{ab}	12.86±2.19^{a}	9.64±2.38^{ab}	15.32±2.87^{a}	8.82±5.10^{ab}	2.16±1.92^{b}	7.93±2.01^{ab}
2-Propenoic acid, 3-phenyl-, ethyl ester	1.3±1.15^{a}	nd	1.35±1.18^{a}	nd	nd	nd	0.66±1.15^{a}	nd
Hexadecanoic acid, ethyl ester	0.6±1.04^{a}	0.74±0.69^{a}	1.04±0.90^{a}	1.18±1.04^{a}	0.98±0.93^{a}	0.8±1.39^{a}	0.84±0.75^{a}	1.49±1.34^{a}
**Total Esters**	**445.25**	**242.74**	**604.32**	**291.89**	**147.14**	**223.6**	**57.79**	**200.31**
Acetic acid	31.88±6.48^{cde}	37.82±14.52^{bcd}	58.59±20.65^{abc}	72.17±16.16^{a}	25.36±4.86^{de}	61.44±2.23^{abc}	3.83±3.42^{e}	67.38±6.48^{ab}
Butanoic acid, 2-methyl	20.5±8.72^{a}	8.2±3.71^{a}	16.25±7.15^{a}	22.18±5.67^{a}	13.21±8.28^{a}	22.74±4.19^{a}	2.6±2.49^{a}	11.82±11.32^{a}
**Total Organic Acids**	**52.38**	**46.02**	**74.84**	**94.35**	**38.57**	**84.18**	**6.43**	**79.2**
2-Butanone-3-hydroxy	29.3±3.72^{ab}	19.17±2.39^{bc}	45.54±13.17^{a}	42.59±1.55^{a}	21.37±4.78^{bc}	18.5±2.43^{bc}	6.32±6.15^{c}	10.94±5.49^{c}
6-Methyl-5-hepten-2-one	5.36±5.59^{a}	5.39±1.28^{a}	7.05±0.72^{a}	7.68±1.38^{a}	8.86±8.61^{a}	6.64±3.33^{a}	4.43±3.93^{a}	4.37±3.88^{a}
**Total Ketones**	**34.66**	**24.56**	**52.59**	**50.27**	**30.23**	**25.14**	**10.75**	**15.31**
Phenol, 2-methoxy-	66.23±12.31^{cde}	33.18±4.92^{e}	42.85±14.83^{de}	33.76±10.58^{e}	143.75±0.42^{ab}	163.08±27.65a	107.64±22.53^{abc}	101.38±43.80^{bcd}
Phenol	8.87±3.82^{a}	4.43±2.68^{a}	5.7±2.207^{a}	4.05±0.84^{a}	10.38±4.77^{a}	16.5±10.12^{a}	6.17±4.71^{a}	7.19±7.51^{a}
Phenol, 4-ethyl-2-methoxy-	nd	nd	nd	nd	nd	nd	2.06±1.793^{a}	2.4±2.74^{a}
Phenol, 4-ethyl-	15.75±2.18^{b}	23.06±8.17^{b}	8.01±2.52^{b}	21.35±1.98^{b}	5.04±1.34^{b}	256.55±105.70^{a}	28.1±10.40^{b}	283.9±106.00^{a}
Vanillin	nd	1.27±1.12^{a}	nd	2.2±2.14^{a}	nd	1.85±1.66^{a}	nd	2.84±0.78^{a}
**Total Phenols**	**90.85**	**61.94**	**56.56**	**61.36**	**159.17**	**437.98**	**143.97**	**397.71**
alfa.-Copaene	54.42±22.9^{ab}	45.15±22.36^{ab}	103.62±23.79^{a}	24.6±34.38^{b}	101.56±23.94^{a}	99.54±6.21^{a}	109.66±20.25^{a}	98.97±41.71^{a}
α-Muurolene,	12.99±4.20^{a}	7.85±0.23^{a}	14.47±2.84^{a}	10.77±1.85^{a}	17.75±3.89^{a}	11.56±2.71^{a}	14.38±3.47^{a}	14.31±6.15^{a}
α-Farnesene	2.05±3.55^{a}	0.91±0.79^{a}	7.23±0.20^{a}	1.4±2.42^{a}	8.08±0.39^{a}	3.36±5.81^{a}	3.58±4.90^{a}	1.78±2.33^{a}
**Total Terpenes**	**69.46**	**53.91**	**125.32**	**36.77**	**127.39**	**114.46**	**127.62**	**115.06**
Dimethyl sulfide	13.47±1.45^{abc}	4.74±2.07^{c}	16.93±6.12^{a}	6.31±2.65^{bc}	14.99±1.81^{ab}	8.73±4.52^{abc}	13.45±1.21^{abc}	7.71±1.38^{bc}

2,3-Butanediol	24.57±42.55[bc]	2.42±4.20[c]	13.87±24.02[bc]	4.6±4.73[bc]	112.41±18.49[a]	12.25±0.94[bc]	65.28±13.33[ab]	18.83±29.77[bc]
Benzofuran, 2,3-dihydro-	2.66±0.45[a]	3.76±2.47[a]	4.89±2.10[a]	3.54±1.07[a]	3.16±1.61[a]	2.77±0.90[a]	2.55±1.11[a]	3.42±0.37[a]
**Total Others**	**40.7**	**10.92**	**35.69**	**14.45**	**130.56**	**23.75**	**81.28**	**29.96**

nd Not detected

a-e Values in the same row with different superscript letters differ significantly ($p < 0.05$) according to one-way ANOVA analysis of variance with a post-hoc Tukey test.
