# Supplementary figures and images for: Metagenetic and Volatilomic Approaches to Elucidate the Effect of Lactiplantibacillus plantarum Starter Cultures on Sicilian Table Olives
Source: Front Microbiol. 2022 Feb 25;12:771636. doi: 10.3389/fmicb.2021.771636 (PMC8914321; doi:10.3389/fmicb.2021.771636)

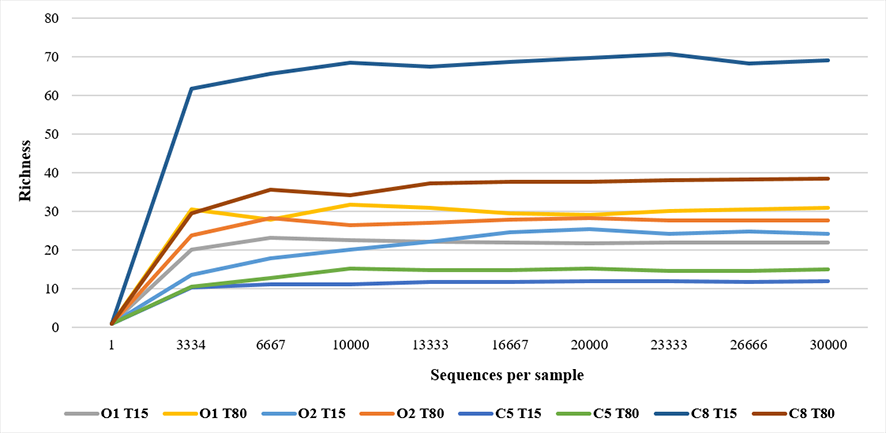

Supplement: Supplementary file 2 [file Image_1.TIF]

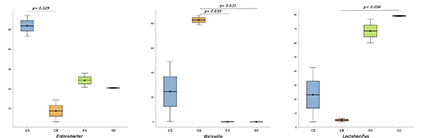

Supplement: Supplementary file 3 [file Image_2.TIFF]

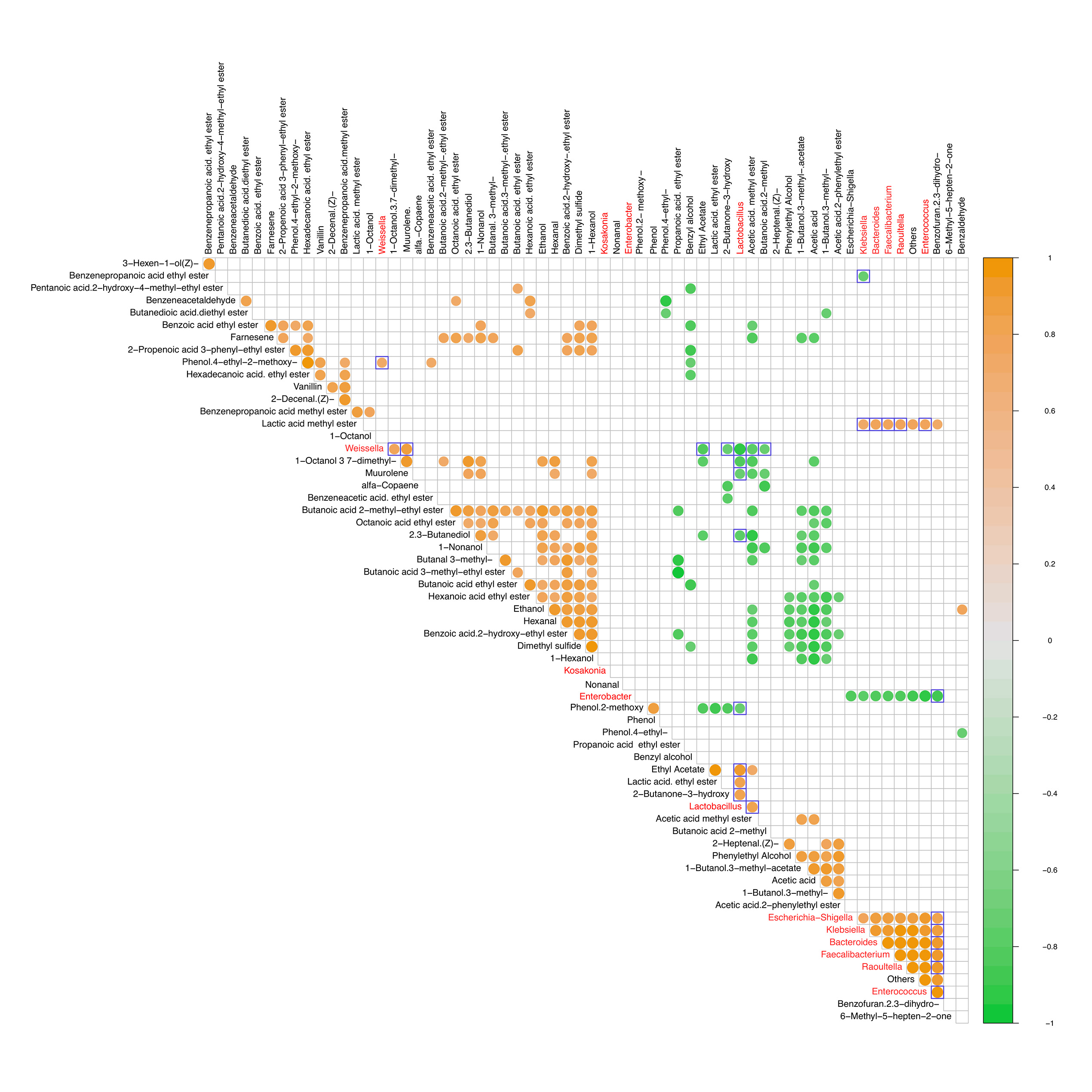

Supplement: Supplementary file 4 [file Image_3.JPEG]
